# Reduced Notch signalling leads to postnatal skeletal muscle hypertrophy in Pofut1^cax/cax^ mice

**DOI:** 10.1098/rsob.160211

**Published:** 2016-09-14

**Authors:** Bilal Al Jaam, Katy Heu, Florian Pennarubia, Alexandre Segelle, Laetitia Magnol, Agnès Germot, Sébastien Legardinier, Véronique Blanquet, Abderrahman Maftah

**Affiliations:** Univ. Limoges, INRA, UMR 1061, UGMA, 87060 Limoges, France

**Keywords:** POFUT1, Notch, satellite cells, skeletal muscles, hypertrophy

## Abstract

Postnatal skeletal muscle growth results from the activation of satellite cells and/or an increase in protein synthesis. The Notch signalling pathway maintains satellite cells in a quiescent state, and once activated, sustains their proliferation and commitment towards differentiation. In mammals, POFUT1-mediated *O*-fucosylation regulates the interactions between NOTCH receptors and ligands of the DELTA/JAGGED family, thus initiating the activation of canonical Notch signalling. Here, we analysed the consequences of downregulated expression of the *Pofut1* gene on postnatal muscle growth in mutant Pofut1^cax/cax^ (cax, compact axial skeleton) mice and differentiation of their satellite cell-derived myoblasts (SCDMs). Pofut1^cax/cax^ mice exhibited muscle hypertrophy, no hyperplasia and a decrease in satellite cell numbers compared with wild-type C3H mice. In agreement with these observations, Pofut1^cax/cax^ SCDMs differentiated earlier concomitant with reduced *Pax7* expression and decrease in PAX7^+^/MYOD^−^ progenitor cells. *In vitro* binding assays showed a reduced interaction of DELTA-LIKE 1 ligand (DLL1) with NOTCH receptors expressed at the cell surface of SCDMs, leading to a decreased Notch signalling as seen by the quantification of cleaved NICD and Notch target genes. These results demonstrated that POFUT1-mediated *O-*fucosylation of NOTCH receptors regulates myogenic cell differentiation and affects postnatal muscle growth in mice.

## 1. Introduction

Skeletal muscles are composed of post-mitotic multinucleated myofibres and satellite cells, which are mononucleated cells located at the periphery of myofibres between the plasma membrane and basal lamina [1]. Satellite cells are precursor stem cells, which are involved in after-birth muscle growth and muscle regeneration after injuries, either by exercise or by disease [2]. These cells are maintained in a quiescent state in normal adult muscles, and upon activation their asymmetric division leads to formation of two cell types: differentiated myogenic cells involved in myofibre formation or repair and self-renewing cells able to replenish the stem pool [3–5]. Postnatal muscle growth can be achieved by either an increase in the number of myofibres (hyperplasia), an increase in their size (hypertrophy), or a combination of both as in rats [6]. In mice, postnatal muscle growth mainly occurs by myofibre hypertrophy and not hyperplasia, with a steady increase in the number of myonuclei per myofibre from birth to three weeks after birth (P21) [7,8]. This myonuclear accretion in myofibres has been shown to be the result of cell fusion between satellite cell-derived myoblasts (SCDMs) and pre-existing myofibres, thus increasing their width and length [9,10]. From P21 to adulthood and thereafter, muscle growth by hypertrophy does not occur by a satellite cell-dependent myogenic fusion [11], but mainly by increased protein synthesis, which provokes an enlargement of myofibres without formation of new myonuclei [8]. Satellite cells are thus the main source of new myonuclei in growing myofibres [12], before their quiescence around P21 [8]. They remain in this state thereafter in normal mature muscle [13], except during regeneration in which satellite cells are re-activated [2].

The IGF/Akt/mTOR and myostatin/Smad pathways are the two major signalling pathways, which control muscle mass increase by hypertrophy during postnatal life after P21, promoting protein synthesis and limiting protein degradation [11]. The genes of the Notch signalling pathway, which are involved in the control of the stem cell's fate and behaviour, are essential for the myogenic progress [14,15]. However, little is known about the role of Notch signalling in perinatal/juvenile muscle growth because Notch1^−/−^ mice mutants [16,17] or embryos lacking components required for Notch signalling such as its main transcriptional repressor RBP-Jk [18] die at midgestation or around birth with severe defects in somitogenesis, vasculogenesis, cardiogenesis and/or neurogenesis [19]. Because global inactivation of Notch signalling leads to drastic and advanced effects on somitogenesis, only conditional mutagenesis of some Notch regulators provides information on the involvement of Notch signalling in myogenesis after midgestation. Indeed, conditional mutations of *Rbp-Jk* [20] and the gene encoding the NOTCH-ligand DLL1 [21] lead to mutant mice exhibiting severe muscle hypotrophy during embryonic development, owing to uncontrolled differentiation of progenitor cells generating a rapid and significant depletion of the progenitor cell pool.

Canonical Notch signalling is initiated by interaction of the extracellular domain of ligands (DLL-1,-3,-4 and JAGGED-1 and -2) with their counterparts on one of the four receptors (NOTCH1–4), leading to sequential proteolytic cleavages by ADAM proteases and the γ-SECRETASE complex of the NOTCH receptor. Once cleaved, the latter releases its NOTCH intracellular domain (NICD), which translocates to the nucleus where it interacts with RBP-Jk by displacing corepressors [22]. This allows the recruitment of coactivators such as MASTERMIND-LIKE-1 (MAML1) [23] to induce transcriptional activation of specific target genes, including *Hes* and *Hey* family genes [24,25]. By activating the expression of target genes such as *Hes1*, Notch inhibits differentiation by repressing the expression of *MyoD* [26], which belongs to the family of myogenic regulating factors (MRFs) including MYF5, MYOGENIN (or MYOG) and MRF4 (or MYF6) [27]. During postnatal muscle growth and muscle regeneration, activated satellite cells coexpress *Pax7* and *MyoD* [28]. While most of them proliferate, myoblasts from activated satellite cells downregulate *Pax7* leading to their differentiation in myocytes, whose fusion gives rise to myogenin-expressing multinucleated myotubes [29]. Some of those proliferating myoblasts (PAX7^+^/MYOD^+^) revert to a quiescent state by repressing *MyoD* expression [30]. Thus, the expression of *Pax7* maintains proliferation and prevents a precocious differentiation, without promoting quiescence [28]. Overexpressed NICD upregulates *Pax7* through a RBP-Jk-dependent binding to its promoter, resulting in enhanced self-renewal of satellite cells, whereas inhibition of Notch signalling leads to a downregulation of *Pax7*, resulting in satellite cell depletion and improved terminal differentiation [30]. Indeed, the loss of *Pax7* expression leads to a complete absence of satellite cells in postnatal skeletal muscles [31].

NOTCH receptors and ligands are glycoproteins, whose extracellular domains are subjected to several glycosylations such as *O*-fucosylation mediated by protein *O*-fucosyltransferase 1 (POFUT1). POFUT1 is a resident *N*-glycosylated glycosyltransferase of the endoplasmic reticulum [32,33], which adds fucose on S or T within the consensus site C^2^X_4_(S/T)C^3^ (where C^2^ and C^3^ are the second and third cysteines, respectively) of epidermal growth factor (EGF)-like repeats present in numerous proteins [34], including NOTCH receptors and ligands. The POFUT1-mediated *O*-fucosylation of extracellular domains of NOTCH receptors controls the receptor–ligand interactions which are critical for activation of Notch signalling [35]. Among the 36 EGF-like repeats of murine NOTCH1 receptor, EGF repeat 12, for example, was necessary and sufficient for ligand binding [36] and mutation of its *O*-fucosylation site affected Notch-ligand interactions [37]. In contrast to NOTCH receptors, *O*-fucosylation of DELTA/JAGGED ligands is poorly documented and recent studies report that *O*-fucosylation of murine DLL1 is not required for ligand–receptor interactions [38]. POFUT1-mediated *O*-fucosylation of receptors is therefore essential for canonical Notch signalling by DLL1 or JAGGED1 [39]. Notch signalling is also modulated by elongation of *O*-linked fucoses on some EGF-like repeats with *N-*acetylglucosamine (GlcNAc) and then with galactose residues, owing to the action of a β1,3-*N*-acetylglucosaminyltransferase of the Fringe family [40] and a β1,4-galactosyltransferase [41,42], respectively. The resulting trisaccharide *O*-fucosylglycan (Galβ1-4GlcNAcβ1-3fucose) can be terminated with α2–3 or α2–6 linked sialic acids, whose involvement in Notch signalling has not been yet reported [43].

In a recent *in vitro* study, we showed that *Pofut1* knockdown reduces Notch signalling and affects differentiation of the mouse myoblast cell line C2C12. The expression patterns of PAX7 and MYOD are modified under these conditions and induce earlier cell differentiation [44]. *In vivo*, knock--out of *Pofut1* is lethal: mice embryos die at E9.5 with a phenotype similar to that of mice in which NOTCH receptor signalling is inactivated [19]. In 2009, a spontaneous mutation in *Pofut1* gene called Pofut1^cax^ was described in a mouse strain [45]. Pofut1^cax/cax^ mice have an insertion of an intracisternal A particle (IAP) in the fourth intron of the *Pofut1* gene, leading to a hypomorphic allele and a decrease in gene expression without any change in protein structure and activity. Homozygous Pofut1^cax/cax^ mice display defects in the axial skeleton consistent with the known patterning functions of Notch in somitogenesis. Nevertheless, no detailed phenotyping was performed on skeletal muscles of Pofut1^cax/cax^ mice.

In this study, we report the consequences of the *Pofut1* hypomorphic mutation on postnatal growth of skeletal muscles in Pofut1^cax/cax^ mice. Immunostaining studies on isolated Pofut1^cax/cax^ skeletal muscles showed a slight but significant muscular hypertrophy with myonuclear accretion compared with wild-type controls. In addition, the number of PAX7^+^ satellite cells was significantly reduced in Pofut1^cax/cax^ mice. Analyses of *ex vivo* Pofut1^cax/cax^ SCDMs revealed a depletion of PAX7^+^/MYOD^−^ progenitor cells, a decrease in *Pax7* expression and disruption of the myogenic programme, leading to earlier Pofut1^cax/cax^ SCDM differentiation. These observations could explain the accrued muscle mass occurring in the first weeks of postnatal life in Pofut1^cax/cax^ mice, as a result of increased fusion of SCDMs with pre-existing myofibres.

## 2. Results

### 2.1. Pofut1^cax^ mutation induces postnatal muscle hypertrophy and decrease in the satellite cell pool

**Figure 1.**
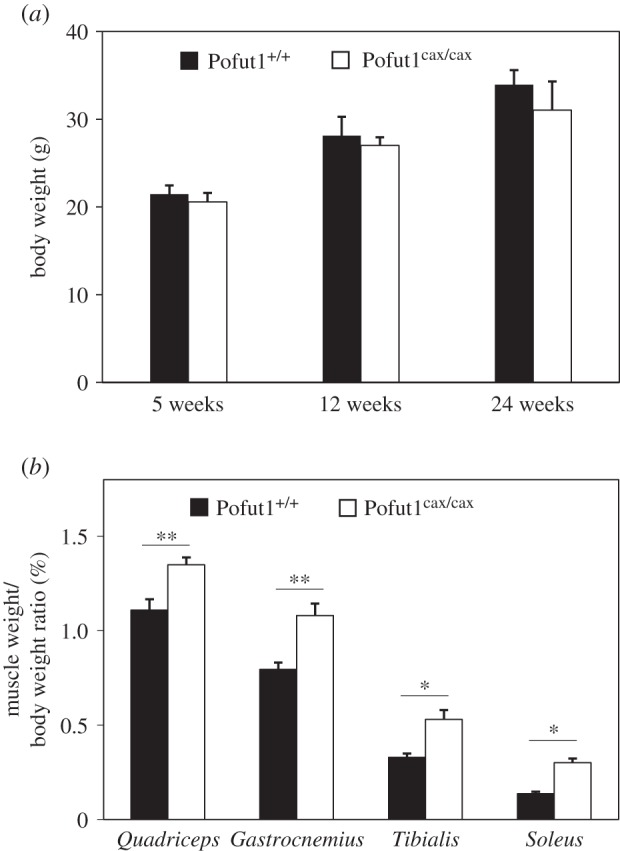
Body weight and muscle/body weight ratios in mice. (*a*) Body weights (g) in Pofut1^+/+^ and Pofut1^cax/cax^ mice (*n* = 6) at three different ages (5, 12, 24 weeks). (*b*) Muscle weight/body weight ratios as a percentage for four different muscles of five week old mice (*n* = 6). Means ± s.e.m. are shown (two-tailed *t*-test, with a significance level of *p < 0.05, **p < 0.01, ***p < 0.001).

As previously described [45], Pofut1^cax/cax^ mice showed either a normal phenotype or shortened bodies with kinky or absent tails. About 40% of Pofut1^cax/cax^ mice had shortened kinky tails (*n* = 19) with a length of 6.16 cm ± 0.68 versus 8.50 cm ± 0.20 in Pofut1^+/+^ mice but showed unchanged body size compared with their wild-type littermates (data not shown). Additional morphometric analyses did not reveal a statistically significant difference (*n* = 6 per genotype and per age) in body weight regardless of the age (5, 12, 24 weeks) of Pofut1^cax/cax^ mice compared with Pofut1^+/+^ mice (figure 1*a*).

**Figure 2.**
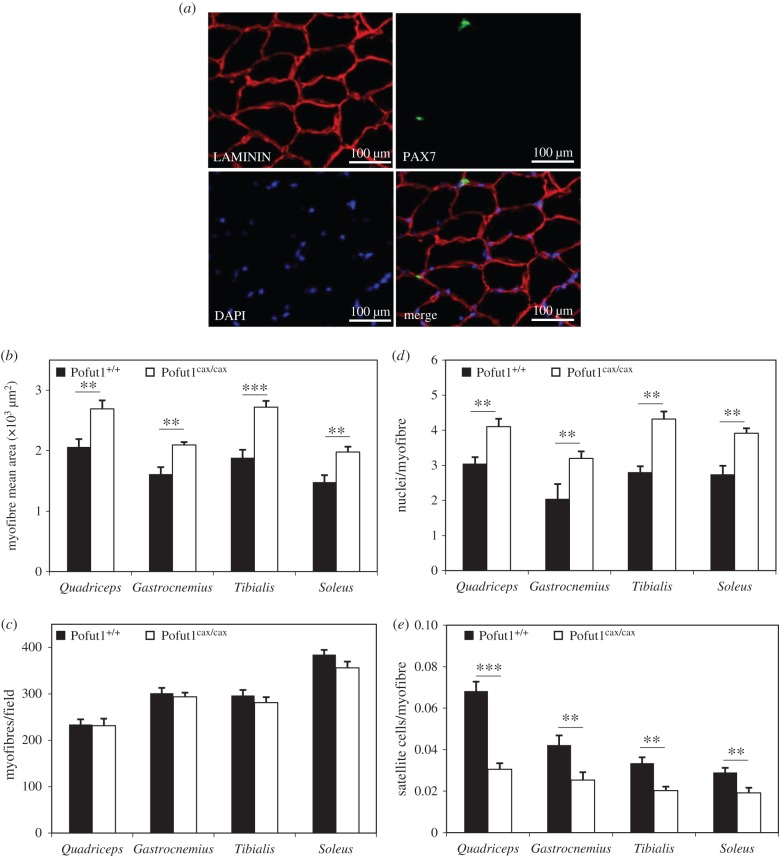
Characterization of immunostained cross sections of skeletal muscles from five week old mice. (*a*) Representative field of *Quadriceps* cryosection from five week old Pofut1^+/+^ mice. LAMININ (red), DAPI (blue) and PAX7 (green) immunostaining showed basal lamina of myofibres, nuclei and satellite cells respectively. (*b*) Myofibre mean areas, (*c*) number of myofibres per field, (*d*) nuclei per myofibre and (*e*) satellite cells per myofibre were determined for skeletal muscles with mixed (*Quadriceps, Gastrocnemius*), fast-twitch (*Tibialis*) or slow-twitch (*Soleus*) myofibres. Means ± s.e.m. (*n* = 6) are shown (two-tailed *t*-test, with a significance level of *p < 0.05, **p < 0.01, ***p < 0.001).

To determine whether the hypomorphic mutation of Pofut1^cax/cax^ mice affected postnatal muscle growth, skeletal muscles with fast-twitch (*Tibialis*) or slow-twitch (*Soleus*) or mixed (*Gastrocnemius* and *Quadriceps*) myofibres were weighed and analysed by immunohistochemistry in Pofut1^cax/cax^ mice and compared with those from wild-type mice after weaning at five weeks (figures 1*b* and 2) and long after sexual maturity at 12 and 24 weeks (electronic supplementary material, table S1). The analysis of muscle weight/body weight (M/B) ratios for these four skeletal muscles from five week old mice revealed higher values in Pofut1^cax/cax^ mice than in Pofut1^+/+^ mice (figure 1*b*). Similar results were found with 12 and 24 week old mice (electronic supplementary material, table S1). These results showed that higher M/B ratios in Pofut1^cax/cax^ mice seem to be unrelated to the type of muscle metabolism and could be due to myofibre hypertrophy, hyperplasia or both. To test these hypotheses, immunohistochemical analyses were carried out to evaluate myofibre mean area and number using cross sections. Immunostaining using anti-laminin antibody (figure 2*a*) showed significant increased myofibre mean area (figure 2*b*) in Pofut1^cax/cax^ mice compared with Pofut1^+/+^ mice but no hyperplasia (figure 2*c*), because the myofibre number per field, although with a lower mean, was not significantly different regardless of muscle type and mouse age (electronic supplementary material, table S1). To determine whether myofibre hypertrophy was only due to increased protein synthesis and/or myonuclear accretion, the number of DAPI-labelled nuclei (figure 2*a*) was evaluated. More nuclei per cross-sectional myofibre were found in each of the four muscles from Pofut1^cax/cax^ mice (figure 2*d*), suggesting greater myonuclear accretion in muscles from Pofut1^cax/cax^ mice.

The pool of satellite cells at the periphery of myofibres was evaluated using anti-Pax7 immunostaining (figure 2*a*) and the number of satellite cells per cross-sectional myofibre for the four selected muscles was determined (figure 2*e* and electronic supplementary material, table S1). Regardless of muscle type and mouse age, the number of satellite cells per cross-sectional myofibre was significantly lower in Pofut1^cax/cax^ mice than in wild-type controls. The decrease in satellite cells ranged from 35% to 66% if considering the four muscles from five week old Pofut1^cax/cax^ mice compared with those from wild-type mice (figure 2*e* and electronic supplementary material, table S1), whereas it was more pronounced (from 33% to 87%) in muscles of 12 and 24 week old mice (electronic supplementary material, table S1).

### 2.2. Pofut1^cax/cax^ satellite cell-derived myoblasts display reduced expression of *Pofut1* and *Pax7*

As the number of Pax7^+^ satellite cells was significantly reduced in Pofut1^cax/cax^ mice compared with the wild-type controls, primary cultures of SCDMs were analysed from skeletal muscles to highlight molecular mechanisms involved in satellite cell depletion.

**Figure 3.**
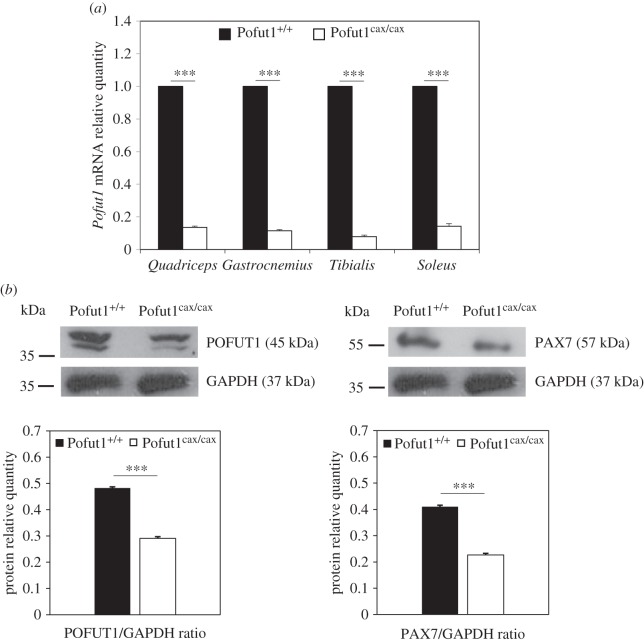
Quantification of *Pofut1* and *Pax7* transcripts and proteins in muscles and SCDMs. (*a*) *Pofut1* mRNA relative quantities in four skeletal muscles from five week old Pofut1^cax/cax^ mice compared with those of Pofut1^+/+^ controls set to 1. Quantities of *Pofut1* mRNA were normalized to *18S RNA* and *Gapdh* mRNA quantities. (*b*) Detection of POFUT1, PAX7 and GAPDH by western blotting on total extracted proteins of proliferating Pofut1^+/+^ and Pofut1^cax/cax^ SCDMs. Histograms represent POFUT1/GAPDH and PAX7/GAPDH ratios. Means ± s.e.m. (*n* = 3) are shown (two-tailed *t*-test, *p < 0.05, **p < 0.01, ***p < 0.001).

Primary cultures of SCDMs from Pofut1^cax/cax^ and Pofut1^+/+^ mice were prepared from a pool of four skeletal muscles of hind legs (*Quadriceps*, *Gastrocnemius*, *Tibialis*, *Soleus*), mixed on the basis of a similar reduction by 80–90% of *Pofut1* mRNA quantities in each of the four selected muscles, as shown in figure 3*a*. As expected, western blot analyses showed a reduction of POFUT1 quantity, thus, validating the *ex vivo* model. This decrease in protein expression was approximately 40% in Pofut1^cax/cax^ SCDMs, when compared with Pofut1^+/+^ SCDMs (figure 3*b*).

Then, expression of PAX7, which plays a key role in activation and quiescence of satellite cells *in vivo*, was evaluated by western blot. A significant 45% reduction in protein expression was obtained for PAX7 in Pofut1^cax/cax^ SCDMs, similarly to POFUT1 (figure 3*b*). These results might suggest that PAX7^+^ satellite cells, less numerous in muscles of Pofut1^cax/cax^ mice, express reduced PAX7 protein levels; this could be consequent to decreased expression of POFUT1.

### 2.3. Pofut1^cax/cax^ satellite cell-derived myoblasts differentiate earlier than wild-type cells

**Figure 4.**
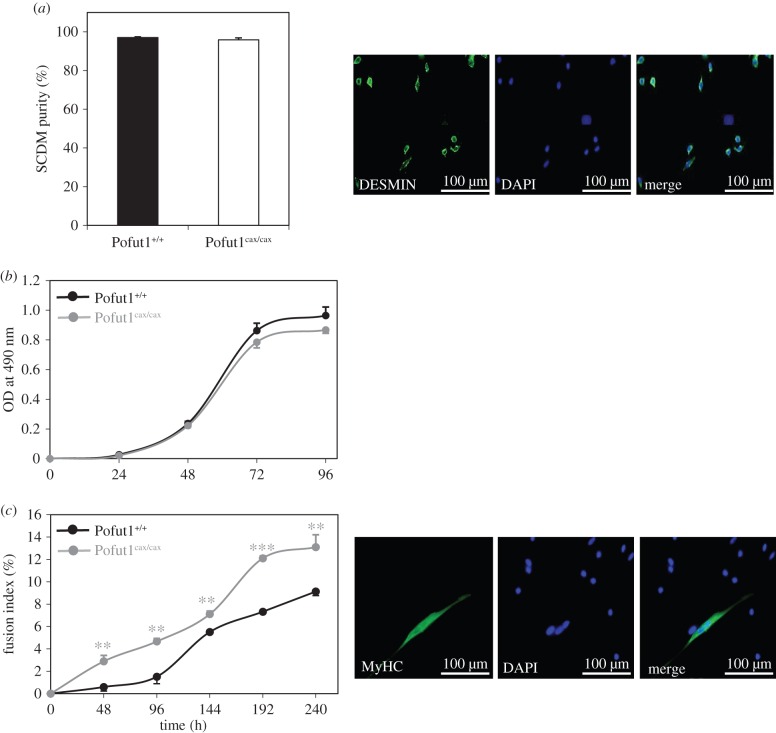
Characterization of purity, proliferation and differentiation of SCDMs. (*a*) Co-immunostaining for DESMIN (green) and DAPI (blue) distinguishes myoblasts from non-muscular cells in primary cultures of Pofut1^+/+^ and Pofut1^cax/cax^ SCDMs. Histogram represents percentage of Desmin^+^ cells related to total DAPI^+^ cells for both SCDM preparations. (*b*) SCDM cell proliferation was determined using an MTS assay, which relies on absorbance of formazan at 490 nm, a product of bioreduced MTS. Test was performed on both SCDMs every 24 h until 96 h after seeding cells at a low density. (*c*) Commitment to differentiation was first evaluated by calculating fusion indexes of Pofut1^+/+^ and Pofut1^cax/cax^ SCDMs. Fusion indexes were determined by immunostaining of nuclei with DAPI (blue) and myotubes with MyHC (green) and were calculated from the percentage of nuclei within MyHC^+^ myotubes compared with total nuclei. Statistical analyses were performed by comparing both SCDMs at each differentiation time (48–240 h). (*a–c*) Data were obtained from three independent experiments in triplicate and error bars indicate standard errors of the mean (two-tailed *t*-test, with a significance level of *p < 0.05, **p < 0.01, ***p < 0.001).

Before analysing *in vitro* differentiation, we determined purity of the myoblast cell population in SCDM preparations by calculating percentage of DESMIN-expressing cells relative to total cells (DAPI^+^). Results showed that primary cultures of Pofut1^+/+^ and Pofut1^cax/cax^ SCDMs contained more than 95% of myoblasts expressing DESMIN (figure 4*a*), which is considered as a muscle-specific protein.

As attested by MTS assay (figure 4*b*), the decreased expression of *Pofut1* did not significantly modify proliferation of Pofut1^cax/cax^ SCDMs from T0 (where cells were seeded at a low density in growth medium (GM)) to 96 h. Indeed, the production of formazan (absorbance at 490 nm) by viable cells is nearly identical for both SCDMs at each time point.

Prior to inducing differentiation by switching the culture medium (differentiation medium (DM) instead of GM), Pofut1^+/+^ and Pofut1^cax/cax^ SCDMs exhibiting comparable purity and proliferation rates were thus seeded at the same cell density. To characterize the differentiation process of both SCDMs, fusion indexes and expression patterns of *Cdkn1a* (cyclin-dependent kinase inhibitor 1a, also called *p21*), *Pofut1*, *Pax7* and three myogenic marker genes (*Myf5, MyoD, Myog*) were determined during 240 h of differentiation. Finally, the impact of decreased *Pofut1* was evaluated by counting the three different cell populations as follows: PAX7^+^/MYOD^−^ (self-renewing cells), PAX7^+^/MYOD^+^ (proliferating cells) and PAX7^−^/MYOD^+^ (differentiating cells).

Following immunostaining of SCDM nuclei and myosin heavy chain (MyHC), the fusion index was calculated by determining the percentage of nuclei in myotubes compared with the total number of nuclei during 240 h of differentiation (figure 4*c*). When seeded at about 50% confluence and placed in DM (T0), Pofut1^cax/cax^ SCDM differentiated significantly faster than Pofut1^+/+^cells with an advance of about 48 h. At 240 h of differentiation, 13% of cell fusion was reached in Pofut1^cax/cax^ SCDMs versus only 9% in wild-type cells. This indicates that the earliest appearance of myotubes in Pofut1^cax/cax^ SCDMs was not due to a difference in proliferation (as demonstrated in figure 4*b*) but more likely to a disruption in the myogenic programme.

**Figure 5.**
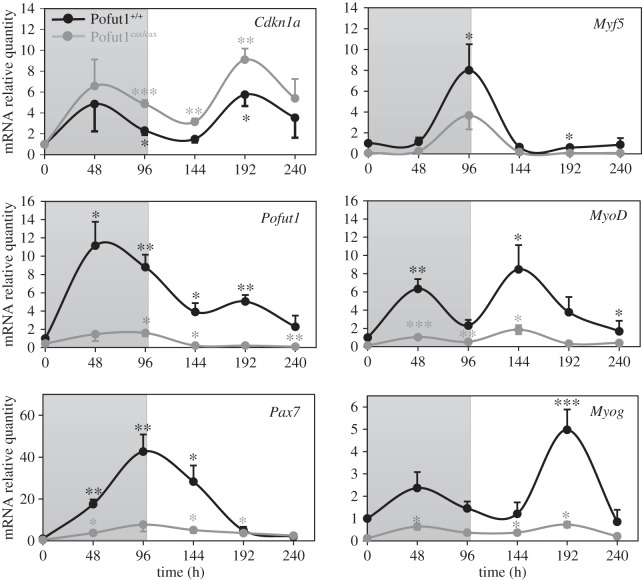
Gene expression for *Cdkn1a*, *Pofut1*, *Pax7* and MRFs during SCDM differentiation. mRNA relative quantities of *Cdkn1a*, *Pofut1, Pax7* and myogenic MRF genes (*Myf5, MyoD and Myog*) in Pofut1^+/+^ and Pofut1^cax/cax^ SCDMs. Area in grey represents the pre-differentiation state. For each cell type, statistical analyses were performed by comparison of each differentiation time point relative to T0. Quantities of target mRNA were normalized to *18S RNA* and *Gapdh* mRNA and then calibrated with T0 using the ΔΔCt method. Data were obtained from three independent experiments in triplicate and error bars indicate standard errors of the mean (two-tailed *t*-test, with a significance level of *p < 0.05, **p < 0.01, ***p < 0.001).

To understand the origin of this premature differentiation, expressions of *Cdkn1a, Pofut1* and *Pax7* as well as of MRFs was followed by qPCR during differentiation for both SCDMs (figure 5). First, differentiation was followed by expression of *Cdkn1a*, a marker for cell cycle arrest. A non-significant variation in the expression of this marker was observed during the first 48 h of differentiation in both SCDMs relative to T0, suggesting that most cells continued to proliferate in spite of induced differentiation. However, its expression was significantly higher at 96 h compared with T0, which might signal a cell cycle exit. This phase (0–96 h), represented in grey in all graphs, was considered as a step where most wild-type cells continued to proliferate. The second phase (96–240 h) where high expression levels of *Cdkn1a*, especially in Pofut1^cax/cax^ SCDMs, were monitored was considered as the main differentiation stage. Globally, expression of *Pofut1*, *Pax7* and MRFs (*Myf5, MyoD, Myog*) was reduced in Pofut1^cax/cax^ SCDMs, compared with the wild-type cells. Expression profiles evolved in the same manner during the differentiation process for cells of the two genotypes, with increases and decreases occurring at the same points of the time course.

In Pofut1^+/+^ SCDM and compared with T0, the expression of *Pofut1*, *Pax7* and *MyoD* was significantly increased at 48 h, whereas that of *Cdkn1a* did not change, in agreement with maintenance of a proliferative state in most cells. The expression of *Pofut1* globally decreased up to the end of differentiation. Interestingly, *Pax7* expression was significantly higher at 96 h compared with T0, whereas that of *MyoD* strongly decreased over time and became not statistically different from T0. From 144 to 192 h, *Cdkn1a* expression increased, as a result of prior induction by *MyoD,* which was optimal at 144 h [46]. Variation of these markers (*Pax7, MyoD, Cdkn1a*) was in favour of a cell cycle exit and commitment of a majority of these cells into differentiation at 96 h. However, as shown by the low fusion index (only 1.5% fusion at 96 h), differentiation was slowed down by the strong expression of *Myf5*. After 96 h, *Myf5* and *Pax7* expression decreased, whereas that of *MyoD* increased up to a maximum at 144 h. Most cells then expressed *Myog* between 144 and 240 h, consistent with fusion of most cells.

In Pofut1^cax/cax^ SCDMs, most cells expressed a significantly higher level of *MyoD* at 48 h as well as at 96 h compared with T0, whereas *Pax7* was weakly expressed at those two time points. While *Pax7* and *Myf5* expression was not significantly high at 96 h, *MyoD* expression was maintained up to 144 h resulting in earlier induction of *Myog*. This is in agreement with a premature commitment to differentiation, as shown by the fusion index.

**Figure 6.**
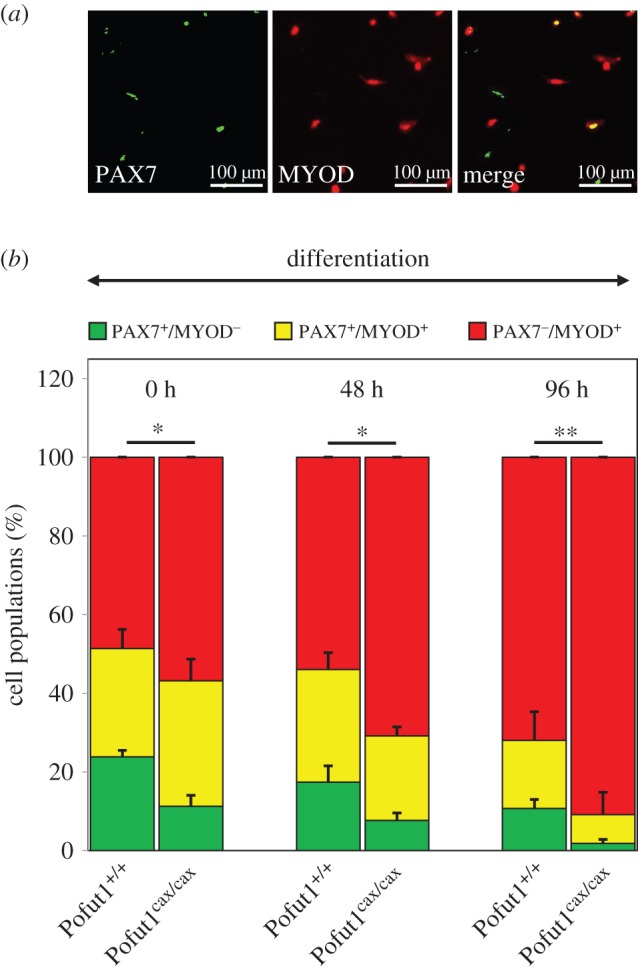
Immunostaining for PAX7 and MYOD during SCDM differentiation. (*a*) Co-immunostaining for PAX7 (green) and MYOD (red) in Pofut1^+/+^ SCDMs. (*b*) Pofut1^+/+^ and Pofut1^cax/cax^ SCDMs were seeded at confluence 4 h before induction of differentiation and then immunostained to distinguish three cell populations: self-renewing cells (PAX7^+^/MYOD^−^), proliferating cells (PAX7^+^/MYOD^+^) and differentiating cells (PAX7^−^/MYOD^+^). Percentages of each population were calculated at three times during differentiation (0, 48, 96 h) and mean ± s.e.m. (*n* = 3) are shown. The significance of the *t*-test (*p < 0.05, **p < 0.01, ***p < 0.001), indicated above histograms for each time, was the same for each analysed cell population.

The transition from proliferative to differentiated state is thus under the control of a *Pax7* and *MyoD* expression equilibrium. In order to further study this transition, SCDMs were seeded at confluence and differentiation was induced 4 h later. We then monitored by immunostaining the expression of PAX7 and MYOD in SCDMs during 96 h of differentiation and counted the three cell populations as follows: PAX7^+^/MYOD^−^, PAX7^+^/MYOD^+^ and PAX7^−^/MYOD^+^ cells (figure 6*a*). During differentiation, the amount of PAX7^+^/MYOD^−^ progenitor cells decreased for the two genotypes in favour of PAX7^−^/MYOD^+^ differentiating cells (figure 6*b*), reflecting commitment of SCDMs to differentiation. Remarkably, the amount of PAX7^+^/MYOD^−^ progenitor cells was drastically decreased by 53%, 56% and 83% in Pofut1^cax/cax^ compared with Pofut1^+/+^ SCDMs at 0 h, 48 h and 96 h, respectively. In parallel, the proportion of PAX7^−^/MYOD^+^ differentiating cells increased.

To conclude, all these results showed an early commitment to differentiation by Pofut1^cax/cax^ SCDMs. Decreased expression of *Pofut1* resulted in a lower *Pax7* expression associated with depletion of PAX7^+^/MYOD^−^ progenitor cells and a lower expression of MRFs in spite of similar expression profiles compared with Pofut1^+/+^ SCDMs.

### 2.4. Pofut1^cax/cax^ satellite cell-derived myoblasts exhibit reduced Notch–ligand interactions leading to lowered Notch signalling

**Figure 7.**
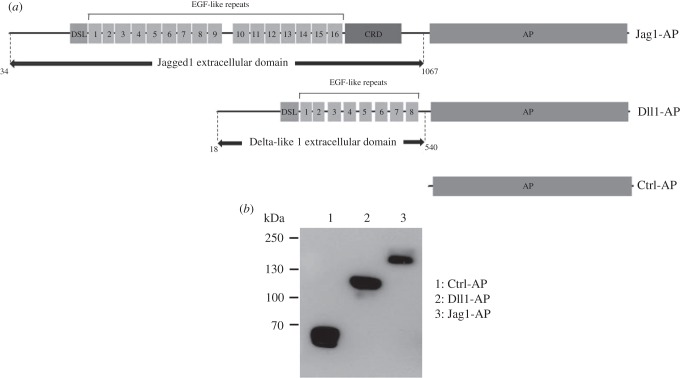
Production of recombinant proteins*.* (*a*) Extracellular domain of murine JAGGED1 (Jag1) and DELTA-LIKE1 (Dll1) were fused to alkaline phosphatase (AP) to obtain recombinant fusion proteins (Jag1-AP and Dll1-AP), which were secreted in culture medium of transfected COS-7 cells, as was the recombinant control protein (Ctrl-AP). EGF-like repeats, Delta-Serrate-Lag domain (DSL) and cysteine-rich domain (CRD) are shown. (*b*) Western blot analysis of recombinant concentrated Ctrl-AP and fusion proteins produced in COS-7 cells, detected with anti-AP antibody.

The Notch signalling pathway members, which control the behaviour and fate of stem cells, are essential for the proper progress of myogenesis [14,15]. POFUT1-mediated *O*-fucosylation of extracellular domains of NOTCH receptors controls receptor–ligand interactions, which are critical for the activation of Notch signalling [35]. In addition, the FRINGE-mediated elongation or not of *O*-linked fucose with GlcNAc residues defines the type of ligands from the DELTA/JAGGED family that can interact with NOTCH receptors [47]. To determine the consequences of decreased *Pofut1* expression in Pofut1^cax/cax^ SCDMs on Notch binding to its ligands, recombinant fusion proteins for two Notch ligands were produced, as previously done for *Drosophila* DELTA [48]. The extracellular domains of murine DLL1 and JAGGED1 including eight and 16 EGF-like repeats respectively were fused to human placental alkaline phosphatase (AP; figure 7*a*). Using an anti-AP antibody, recombinant Ctrl-AP, Dll1-AP and Jag1-AP from concentrated supernatants of transfected COS-7 cells were detected at about 60, 120 and 175 kDa, respectively (figure 7*b*). These apparent molecular weights were higher than expected on the basis of amino acid sequences (54.6, 109.9 and 166.8 kDa, respectively). These results suggested the presence of posttranslational modifications such as *N*- and *O*-glycosylations, as predicted by *in silico* analyses and known for the ligands [43].

**Figure 8.**
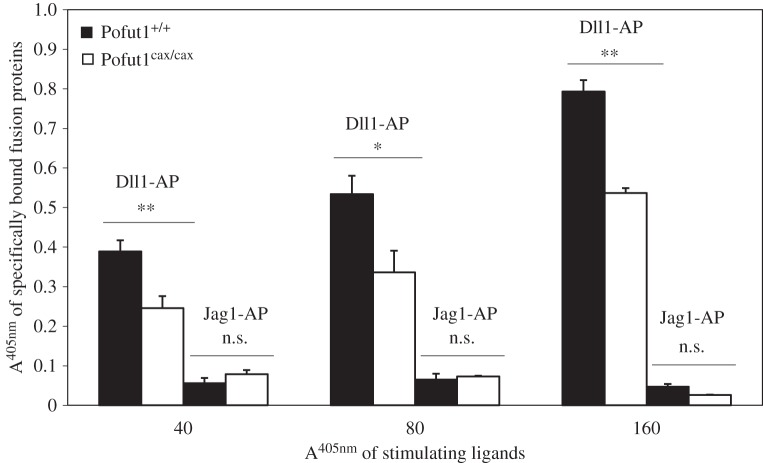
Binding assays of recombinant fusion proteins at the cell surface of proliferating SCDMs*.* Specific binding of Dll1-AP and Jag1-AP at cell surfaces of proliferating Pofut1^+/+^ and Pofut1^cax/cax^ suspension cells of SCDMs. AP assays based on absorbance at 405 nm (A^405 nm^) of pNPP substrate were performed on cell lysates after incubation of SCDM suspensions with three increasing doses of concentrated recombinant proteins (Dll1-AP, Jag1-AP or Ctrl-AP), tested as stimulating ligands and quantified on the basis of their AP activity. Absorbance values of Ctrl-AP were subtracted. Mean absorbance values ± s.e.m. (*n* = 3) were compared between both types of SCDMs for each fusion protein (two-tailed *t*-test, *p < 0.05, **p < 0.01, ***p < 0.001, n.s., not significant).

**Figure 9.**
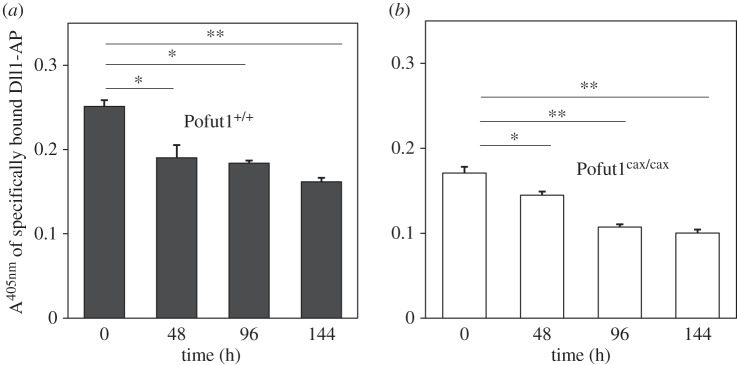
Quantification of specific binding of Dll1-AP at cell surface of SCDMs during differentiation*.* The specific binding of Dll1-AP at cell surfaces of Pofut1^+/+^ (*a*) and Pofut1^cax/cax^ (*b*) was determined every 48 h until 144 h of differentiation, using adherent SCDMs seeded at confluence. AP assays based on absorbance at 405 nm (A^405 nm^) of pNPP substrate were performed on cell lysates after incubation of SCDMs with unconcentrated proteins (Dll1-AP or Ctrl-AP), quantified on the basis of their AP activity. Absorbance values of Ctrl-AP were subtracted to determine binding specificity of Dll1-AP. Mean absorbance values ± s.e.m. (*n* = 3) were compared with those at T0 for each cell type (two-tailed *t*-test, *p < 0.05, **p < 0.01, ***p < 0.001).

Proliferating Pofut1^+/+^ and Pofut1^cax/cax^ SCDMs were incubated simultaneously for 1 h 30 with the same quantities of recombinant proteins (Ctrl-AP, Dll1-AP or Jag1-AP) at three different doses, corresponding to an absorbance at 405 nm of 40, 80 or 160. Proteins were previously concentrated by ultrafiltration from supernatants of transfected COS7 cells and quantified on the basis of AP activity. The specific binding of these proteins to NOTCH receptors on SCDMs was determined using an AP assay by subtracting Ctrl-AP activity (background). For both SCDMs, Dll1-AP binds specifically to cells and its binding increased in a dose-dependent manner, while binding of Jag1-AP was very low (A^405 nm^ < 0.08), regardless of the dose of stimulating ligand (figure 8). Moreover, the specific binding of Dll1-AP was significantly decreased in Pofut1^cax/cax^ SCDMs, varying between −32% and −37% according to the dose. Because binding of Jag1-AP on 200 000 cells was very low and did not significantly increase according to the dose of ligand, it could not be considered as specific. For this reason, only binding of Dll1-AP was followed during 144 h of SCDM differentiation (figure 9). To avoid centrifugation during multiple washing steps that could prevent optimal binding of recombinant proteins, confluent adherent SCDMs were seeded in multi-well plates and incubated with the same dose of recombinant proteins (Dll1-AP, Ctrl-AP) diluted in HBAH at the same concentrations on the basis of their AP activity. A significant reduction in specific Dll1-AP binding was clearly observed in both SCDMs during the time course; this result is likely to be related to the reduction in *Pofut1* expression during differentiation (figure 5).

**Figure 10.**
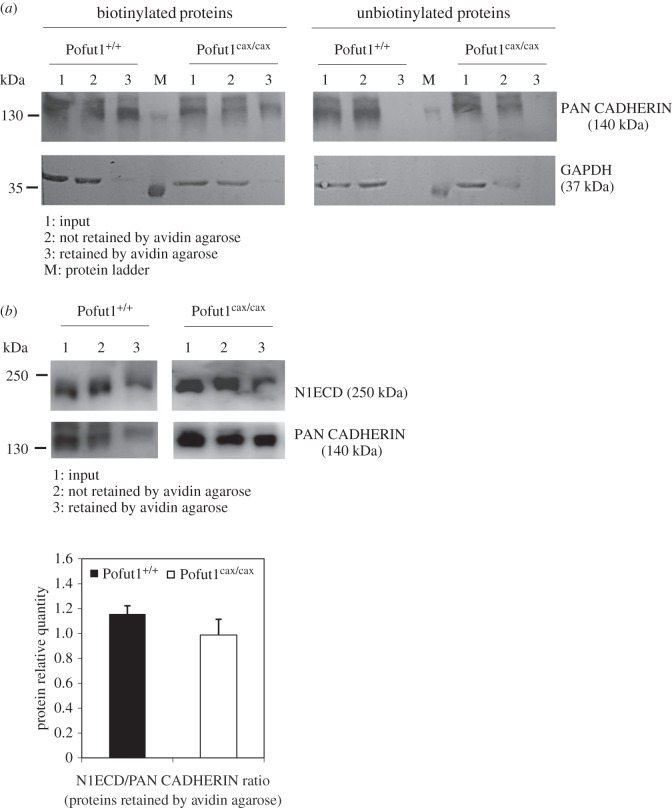
Quantification of NOTCH1 receptors at the cell surface of SCDMs. (*a*) Western blot using an antibody directed against members of the cadherin family (PAN-CADHERIN), which are membrane proteins and not considered as target proteins of POFUT1. PAN-CADHERIN and GAPDH were monitored during purification of cell surface membrane proteins on avidin agarose after biotinylation or not of scraped SCDMs. Purified fractions containing initial extracted proteins (input. lanes 1), unretained proteins (lanes 2) or proteins eluted from avidin agarose (lanes 3) were analysed for their content of PAN-CADHERIN and GAPDH. (*b*) After cell surface biotinylation of proliferating Pofut1^+/+^ and Pofut1^cax/cax^ SCDMs and purification of biotinylated surface proteins using avidin agarose, all fractions described above were analysed by western blot using antibodies against NOTCH1 extracellular domain (N1ECD) and PAN-CADHERIN. Histograms represents the N1ECD/PAN-CADHERIN ratio, determined after quantification of these two proteins in lanes 3. Means ± s.e.m. (*n* = 3) are shown (two-tailed *t*-test, *p < 0.05, **p < 0.01, ***p < 0.001).

Because decreased ligand–receptor interactions in Pofut1^cax/cax^ proliferating SCDMs (figure 8) could be due to lower amounts of NOTCH receptors at the cell surface and/or to reduced POFUT1-mediated *O*-fucosylation of NOTCH receptors, the quantity of NOTCH1 receptors present at the cell surface of SCDM was determined. The amount of biotinylated extracellular domain of NOTCH1 receptor (N1ECD) retained by avidin agarose was reported relative to the amount of PAN CADHERIN (figure 10). First, we showed that the antibody raised against PAN-CADHERIN (recognition of all cadherin members) detected specific bands at about 140 kDa in the pool of proteins eluted from avidin agarose (figure 10*a*, lanes 3) only when proteins (input) were previously biotinylated (figure 10*a*, lanes 1). The cytosolic GAPDH protein was mainly detected for the input and not retained protein samples (figure 10*a*, lanes 1 and 2). Only a small amount of GADPH was found in the eluted sample (figure 10*a*, lanes 3), showing that biotinylated proteins, retained by avidin–agarose, were mostly membrane proteins expressed at the cell surface.

After SCDM cell surface biotinylation and capture of biotinylated proteins by avidin agarose, N1ECD and PAN-CADHERIN were specifically detected by western blot (figure 10*b*, lanes 1–3) in all the samples (input, unretained proteins and proteins eluted from avidin agarose). N1ECD/PAN-CADHERIN ratios were similar in Pofut1^+/+^ and Pofut1^cax/cax^ SCDMs, demonstrating that the decrease in ligand–receptor interactions in Pofut1^cax/cax^ SCDM is in fact the consequence of lower *O*-fucosylation of NOTCH receptors.

**Figure 11.**
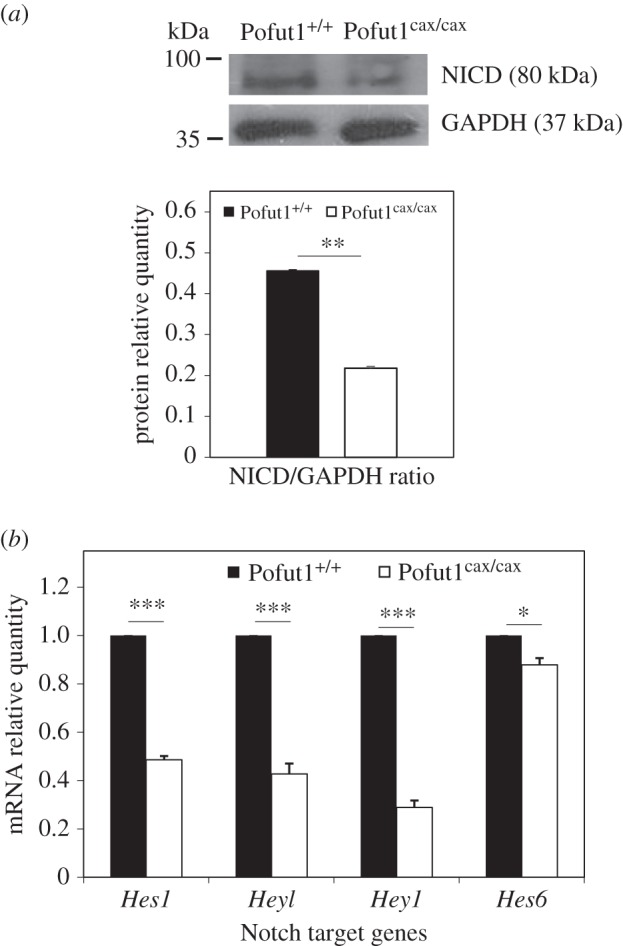
Notch signalling pathway activation. (*a*) Detection of cleaved NOTCH intracellular domain (NICD) and GAPDH by western blotting on total extracted proteins of proliferating Pofut1^+/+^ and Pofut1^cax/cax^ SCDMs. Histograms represent Notch signalling activation (calculated by cleaved NICD/GAPDH ratio). (*b*) Gene expression levels of Notch target genes (*Hes1*, *Heyl*, *Hey1*) and *Hes6* during proliferation. Target mRNAs were normalized to *18S RNA* and *Gapdh* mRNA. Means ± s.e.m. (*n* = 3) are shown (two-tailed *t*-test, *p < 0.05, **p < 0.01, ***p < 0.001).

To study the effects of decreased *Pofut1* expression on Notch signalling, relatively cleaved NICD quantities in total protein extracts from Pofut1^+/+^ and Pofut1^cax/cax^ SCDMs were compared by western blot (figure 11*a*) because they reflect NOTCH cleavage after ligand binding to NOTCH receptors. Results showed a significantly lower cleaved NICD/GAPDH ratio of approximately 50% in Pofut1^cax/cax^ SCDMs compared with wild-type cells, consistent with reduced interaction of NOTCH receptors with Dll1-AP.

Furthermore, we analysed the expression of three members of the Hes/Hey family (*Hes1*, *Hey1*, *Heyl*), which are recognized as target genes of Notch and regulated by NICD [49] and *Hes6*, which does not appear to be regulated by the Notch pathway [50]. Expression levels of *Hes1*, *Hey1* and *Heyl* were significantly reduced (50–75%) in Pofut1^cax/cax^ SCDMs, whereas *Hes6* expression was slightly less (figure 11*b*). Together, these results reflect the reduction in Notch signalling, induced by decreased *Pofut1* expression in Pofut1^cax/cax^ SCDMs.

## 3. Discussion

*Pofut1*-null mouse embryos exhibit severe retarded growth and die at midgestation (≈E10) with major defects in many developmental processes such as somitogenesis and neurogenesis, owing to a global inactivation of Notch signalling [19]. Pofut1^cax/cax^ mice are characterized by decreased expression of *Pofut1* mRNA and protein, resulting in reduced Notch signalling [45]. These mice with hypomorphic *Pofut1* alleles are viable and fertile but exhibit skeletal defects of variable intensity, affecting the length and shape of the body and tail, subsequent to defects in somitogenesis. Our present data show for the first time that the downregulated expression of *Pofut1* also affects postnatal muscle growth in mice leading to a moderate muscular hypertrophy of skeletal muscles with myonuclear accretion and a significant decrease in the satellite cell pool. We did not observe any hyperplasia in muscles from Pofut1^cax/cax^ mice, regardless of mouse age. This result is in agreement with previous studies showing that postnatal muscle growth in mice occurs by hypertrophy and not hyperplasia [7], because the myofibre number is already established around birth in many muscles [51].

All satellite cells of skeletal muscles which express *Pax7* derived from PAX3^+^/PAX7^+^ muscle progenitor cells of central dermomyotome [52]. They adopt their satellite position just before birth (E16.5–18.5) [53,54]. Once this first pool of satellite cells is constituted, their number per myofibre steadily decreases from P6 to reach the adult level at P21, whereas the number of myonuclei steadily increases during the same period in murine *extensor digitorum longus* muscles [8], which contains slow-twitch myofibres such as the *Soleus* muscle used in our study. After P21 in mouse, the number of satellite cells and myonuclei is relatively stable in the physiological state and muscle growth occurs essentially by protein anabolism resulting in a myofibre volume increase without satellite cell activation and addition of new myonuclei. After weaning, in five week old mice (P35), we showed that all skeletal muscles from Pofut1^cax/cax^ mice displayed myofibre hypertrophy with myonuclear accretion but less satellite cells compared with wild-type mice. Consequently, we hypothesize that the number of satellite cells decreased faster in muscles of Pofut1^cax/cax^ mice between P6 and P21, at the same time as a more rapid increase in the number of post-mitotic myonuclei as a result of more myoblast fusion related to hypertrophy. However, we cannot rule out that a reduced number of PAX7^+^ satellite cells is already present at birth in Pofut1^cax/cax^ mice compared with Pofut1^+/+^ controls.

In Pofut1^+/+^ and Pofut1^cax/cax^ mice, myofibre mean areas of most skeletal muscles continued to significantly increase between 5 and 12 weeks of age in the same proportions. It then became stable between 12 and 24 weeks except for *Quadriceps*. These results suggest that IGF/Akt/mTOR and myostatin/Smad pathways that govern the postnatal muscle growth by increased protein synthesis from P21 to adulthood were not significantly affected by reduced Notch signalling in Pofut1^cax/cax^ mice. However, Notch signalling may affect the IGF/Akt/mTOR pathway, by regulating the transcription of the gene encoding the phosphatase PTEN, which prevents activation of Akt (also named protein kinase B) by dephosphorylating PIP3 [55]. Indeed, Notch signalling leads to an activation of target genes *Hes1* and *c-Myc*, which act as transcriptional repressors of *Pten* expression [56]. Conversely, RBP-Jk-dependent Notch signalling induces *Pten* upregulation [55,57,58]. It is therefore difficult to predict how downregulation of *Pofut1* which leads to reduced Notch signalling might impact IGF/Akt/mTOR pathway through regulation of the *Pten* expression. However, it was previously shown that inhibition of Notch signalling by DAPT treatment induces myotube hypertrophy, through myostatin decrease that promotes activation of the Akt pathway [59].

Notch signalling regulates myogenic differentiation and satellite cell fate [15]. Primary cultures of SCDMs were settled from skeletal muscles of Pofut1^cax/cax^ mice to better understand the origin of postnatal muscular hypertrophy. First, we showed comparable purities and proliferation rates of SCDM preparations and validated the decreased expression of *Pofut1* in Pofut1^cax/cax^ SCDMs, compared with wild-type SCDMs. We also showed an earlier commitment to differentiation of Pofut1^cax/cax^ SCDMs, subsequent to a global decrease in Notch signalling provoked by reduced amounts of POFUT1. Indeed, reduced POFUT1 expression was directly correlated with reduced binding of the Dll1-AP fusion protein to NOTCH receptors at the cell surface of Pofut1^cax/cax^ SCDMs, leading to a lesser amount of cleaved NICD and reduced expression of several Notch target genes from the Hes/Hey family and Pax7 [30]. Our results strongly suggest that reduction of these ligand–receptor interactions was only due to decreased POFUT1-mediated *O*-fucosylation of NOTCH receptors, because the relative quantity (RQ) of NOTCH receptors expressed at the cell surface was unaffected in Pofut1^cax/cax^ SCDMs, as also previously observed in mouse C2C12 myogenic cell knockdown for *Pofut1* [44]. This might strengthen the hypothesis that POFUT1 does not have a chaperone role for mammalian NOTCH receptors, in contrast to what had been previously described in *Drosophila* [60]. However, only NOTCH1 receptors were quantified at the cell surface out of the four mammalian ones.

Among 20 predictive *O*-fucosylation sites within the mouse NOTCH1 extracellular domain composed of 36 EGF-like repeats [35], more than 50% of sites are known to be really modified by *O*-linked fucoses [61]. Recently, the majority of predicted *O*-fucosylation sites were shown to be occupied on *Drosophila* Notch by fucose residues, whose degree of elongation by Fringe was variable [62]. Thus, even though NOTCH receptors would have less *O*-fucosylated EGF-like repeats in Pofut1^cax/cax^ SCDMs than in wild-type cells, we cannot rule out that some EGF-like, such as the highly conserved EGF-like repeat 12 [36], are *O*-fucosylated in priority and even elongated by Fringe owing to their involvement in modulation of Notch receptor–ligand interactions.

We showed that both proliferating SCDMs specifically bound Dll1-AP to their cell surfaces in a dose-dependent manner, whereas the very low binding of Jag1-AP was not considered as relevant. This might be related to a high activity of glycosyltransferases of the Fringe family, which allow GlcNAc extension of *O*-linked fucose residues on NOTCH EGF repeats and thus promote binding to Delta and alter binding to Jagged, as previously shown [63]. In addition, we showed a gradual decrease in Dll1-AP binding during differentiation in both SCDMs, which could be explained by a gradual reduced expression of *Pofut1* throughout this process.

As in *Pofut1* knockdown C2C12 cell line (called Po−) [44], we showed earlier differentiation of Pofut1^cax/cax^ SCDMs owing to reduced Notch signalling and a significant lowering in PAX7^+^/MYOD^−^ progenitor cells in favour of more PAX7^−^/MYOD^+^ cells committed to differentiation. Thus, *in vitro* and *ex vivo* cell models represented by the C2C12 Po− cell line and Pofut1^cax/cax^ SCDMs, respectively, gave similar results, highlighting the essential role of POFUT1 in myogenic differentiation through Notch signalling regulation.

While NOTCH receptors and their canonical ligands are already known targets of POFUT1, other proteins could be directly or indirectly affected by the decreased expression of *Pofut1* gene in Pofut1^cax/cax^ mice. Indeed, approximately 100 proteins are predicted to be *O*-fucosylated [34], some of which might be involved in one of the main signalling pathways responsible for postnatal muscle growth. Except by mass spectrometry, it is difficult to demonstrate that a natural protein is actually *O*-fucosylated as no antibody or specific lectin recognizing *O*-linked fucose residues is available and no enzyme is known to specifically cleave O-fucose. Thus, it is experimentally difficult to know which sites are precisely *O*-fucosylated on NOTCH receptors expressed at the cell surface of SCDMs and how decreased expression of *Pofut1* could affect *O*-fucosylation of these sites.

Finally, it would be of interest to test the impact of a more drastic downregulation of *Pofut1* on postnatal muscle growth of heteroallelic Pofut1^null/cax^ mice and to analyse the functional consequences of muscular hypertrophy on muscle strength and locomotor activity in these murine models.

## 4. Material and methods

### 4.1. Animals

Pofut1^cax^ is a spontaneous mutation on a C3H background, which results from the insertion of an IAP retrotransposon into the fourth intron of the *pofut1* gene [45]. Mice were maintained under this background by intercrossing Pofut1^+/cax^ animals. Pofut1^+/+^ and Pofut1^cax/cax^ mice were genotyped from genomic DNA by PCR as previously described [45]. All mice were bred and housed in the animal facility of Limoges University under controlled specific pathogen free conditions (21°C, 12 h light/12 h dark cycle, environmental enrichment) with free access to standard mouse chow and tap water.

### 4.2. Isolation of satellite cell-derived myoblasts

Primary cultures of myoblasts were obtained from mice of five weeks of age. SCDMs were isolated from skeletal muscles of hind legs after enzymatic digestion by pronase, as previously described [64]. Cells were plated at a density of 15 000 cells cm^−2^ on Matrigel^®^-coated Petri dishes (BD Biosciences, Franklin Lakes, NJ) in Ham's F10 (Invitrogen, Thermo Fisher Scientific, Waltham, MA) supplemented with 20% horse serum, 100 units ml^−1^ penicillin and 100 µg ml^−1^ streptomycin. Cells were maintained for 48 h at 37°C and 5% CO_2_ then washed with Ham's F10 before being placed in the GM, i.e. Ham's F10 supplemented with 5 ng ml^−1^ basic fibroblast growth factor (bFGF, Invitrogen, Thermo Fisher Scientific), 20% heat-inactivated horse serum, 100 units ml^−1^ penicillin and 100 µg ml^−1^ streptomycin. The myoblast population was enriched by eliminating fast-adherent fibroblasts using serial 30 min preplating procedures. To induce differentiation, SCDMs were incubated at 40–50% confluence in a DM consisting of Ham's F10 containing 10% heat-inactivated horse serum, 100 units ml^−1^ penicillin and 100 µg ml^−1^ streptomycin. For estimation of MYOD and/or PAX7 expressing cell populations and for binding assays during the time course of differentiation, SCDMs were seeded at confluence and differentiation was induced four hours later. SCDM purity was determined by the proportion of cells expressing DESMIN (an intermediate filament protein located in proliferating skeletal myoblasts) compared with total cell number (DAPI+ cells).

### 4.3. Proliferation

SCDMs were seeded at 5000 cells per well in GM into 96-well plates and 20 µl of MTS (Cell Titre 96 Aqueous Non-Radioactive cell proliferation assay; Promega Corp., Madison, WI) in a 200 µl final volume were added at 0, 24, 48, 72 and 96 h. The plates were then incubated for 1 h at 37°C and absorbance of formazan, a product from the bioreduced MTS, was measured at 490 nm using an ELISA plate reader (FLUOstar Omega; BMGLabtech, Ortenberg, Germany). Six replicates were analysed at each time point and absorbance values of background (GM) were subtracted.

### 4.4. Cell culture and transient transfections of COS-7 cells

COS-7 cells (ATCC-CRL 1651) were maintained at 37°C in DMEM supplemented with 10% heat-inactivated fetal bovine serum, 100 units ml^−1^ penicillin and 100 µg ml^−1^ streptomycin. COS-7 cells were seeded in 100 mm tissue culture dishes and transfected at 75% confluence using 18 µl of XtremeGene9 transfection reagent (Roche Applied Science, Mannheim, Germany) with 6 µg of each construct in serum-free DMEM supplemented with 1% penicillin/streptomycin. Supernatants were recovered by centrifugation at 96 h after transfection.

### 4.5. Ligand constructs

Dll1-AP and Jag1-AP were constructed by cloning the sequences encoding amino acids 1–540 of mouse DELTA-LIKE PROTEIN 1 (NP_031891.2) and amino acids 1–1067 of mouse JAGGED1 (NP_038850.1), respectively, in frame with human placental AP from pAPtag-2 (GenHunter Corp., Nashville, TN). Total cDNA from mouse tissues was used as a template for PCR to generate modified cDNA ends with *Hind*III site in 5′ and *Bgl*II or *Bam*HI site in 3′. After subcloning into pGEM-Teasy (Promega Corp.) and enzymatic digestion with appropriate restriction enzymes, purified fragments were directly cloned into pAPtag-2 using *Hind*III and *Bgl*II cloning sites to obtain the constructs referred to as pAPtag2-Dll1 and pAPtag2-Jag1. According to a previously described cloning technique [65], the pAPtag-2 was modified with prehybridized oligonucleotides encoding a signal peptide promoting secretion of recombinant Ctrl-AP. Eight prehybridized oligonucleotides encoding the same Igκ-chain leader sequence as found in commercial pSecTag/FRT/V5-His-TOPO (Invitrogen, Thermo Fisher Scientific) were cloned between *Hind*III and *Bgl*II cloning sites downstream from the cytomegalovirus promoter in pAPtag-2. The plasmidic constructs were sequenced and were used to transiently transfect COS-7 cells.

### 4.6. RNA extraction, reverse transcription and gene expression analysis

Total RNA was extracted from SCDMs and hind limb skeletal muscles of five week old Pofut1^+/+^ and Pofut1^cax/cax^ mice using RNeasy mini kit (Qiagen, Inc., Hilden, Germany). Quality and quantity of total RNA were measured using an Agilent 2100 bioanalyzer (Santa Clara, CA) and a Nanodrop 1000 spectrophotometer (Wilmington, DE), respectively. The high capacity cDNA reverse transcription kit (Invitrogen, Thermo Fisher Scientific) was used to convert 2 µg of total RNA into single-stranded cDNA. Semi-quantitative PCR was performed from 2 ng total cDNA in an ABI Prism 7900 Sequence Detector System (Applied Biosystems, Thermo Fisher Scientific) using 40 cycles at 95°C for 15 s followed by 60°C for 1 min. Taqman^®^ primers and probe sets used in this study were as follows: 18S (Hs99999901_s1), Gapdh (Mm99999915_g1), Cdkn1a (Mm00432448_m1), Pofut1 (Mm00475567_m1), Pax7 (Mm03053796_s1), MyoD1 (Mm00440387_m1), Myog (MM00446194_m1), Myf5 (Mm00435125_m1), Hes1 (Mm00468601_m1), Heyl (Mm00516555_m1), Hey1 (Mm00468865_m1) and Hes6 (Mm00517097_g1). As described previously [44], the ΔΔCt method was used to quantify the relative abundance of each mRNA. RQ values, calculated only for a threshold cycle (Ct) lower than 37, reflected expression changes in the sample of interest compared with the calibrator sample, after normalization with *18S* and *Gapdh* reference genes. Statistical analyses were performed by comparison of each differentiation time (from 48 to 240 h) relative to T0 for wild-type cells, which was set as 1. Expression of *Pofut1* in different skeletal muscles (*Quadriceps*, *Gastrocnemius*, *Tibialis* and *Soleus*) from Pofut1^cax/cax^ mice was also studied, using wild-type muscles as a calibrator.

### 4.7. Protein extraction and western blot

Proteins were isolated from SCDMs with RIPA extraction buffer (50 mM Tris–HCl, 150 mM NaCl, 0.5% sodium deoxycholate, 1% NP-40, 0.1% SDS, pH 8) containing a protease inhibitor cocktail (Roche Applied Science). Protein lysates were then centrifuged at 12 000*g* for 10 min at 4°C and soluble proteins from supernatants were quantified using a bicinchoninic acid protein assay (Sigma-Aldrich Corp., St Louis, MO) with bovine serum albumin (BSA) as a standard. Equal amounts of extracted proteins (30–100 µg) were separated under denaturing and reducing conditions on SDS–polyacrylamide gels (6–12%) and then transferred to a Hybond C-extra nitrocellulose membrane (GE Healthcare, Buckinghamshire, UK). Then, membranes were blocked using 5% non-fat dried milk (w/v) in TBST (50 mM Tris–HCl, 150 mM NaCl, 0.1% Tween-20, pH 7.4) for 1 h at room temperature, followed by incubation overnight at 4°C with specific primary antibodies diluted in 2.5% non-fat dried milk (w/v) in TBST. The following primary antibodies were used for immunoblotting: 1 : 500 dilution of anti-Pofut1 purified antibody used in a previous study [44], 1 : 50 dilution of anti-Pax7 antibody (Developmental Studies Hybridoma Bank, University of Iowa, IA), 1 : 2000 dilution of anti-GAPDH (R&D Systems Inc., Minneapolis, MN), 1 : 300 dilution of anti-cleaved NOTCH1 (Val1744; Cell Signaling Technology, Danvers, MA). After three washes in TBST, membranes were incubated for 1 h at room temperature with 1 : 1000 dilution of secondary HRP conjugate antibodies (Dako, Glostrup, Denmark) in 2.5% non-fat dried milk (w/v) in TBST. After three more washes in TBST, immunoblots were developed using BM Chemiluminescence western blotting substrate (peroxidase, POD; Roche Applied Science) and exposed (Hyperfilm ECL, GE Healthcare). For relative quantification, analysis of band intensities was carried out using ImageJ software (Wayne Rasband, National Institutes of Health, USA).

### 4.8. Production, quantification and characterization of recombinant proteins

Recombinant proteins (Ctrl-AP, Dll1-AP and Jag1-AP) from harvested supernatants of transfected COS-7 cells were directly used for binding assays or concentrated 25- to 30-fold by ultrafiltration in binding buffer HBAH (Hanks' balanced salt solution, 0.5 mg ml^−1^ BSA, 0.1% sodium azide, 20 mM Hepes, pH 7). To estimate protein quantities, we quantified the enzymatic activity of AP by measuring the absorbance of para-nitrophenylphosphate, whose dephosphorylation led to a coloured product, according to recommendations of GenHunter (GenHunter Corp., Nashville, TN). The integrity of concentrated fusion protein and Ctrl-AP was checked by western blot using a 1 : 1000 dilution of anti-AP antibody (GenHunter Corp.). For AP assay, 120 µl pure or diluted protein samples were added to 120 µl of 2X AP reagent (2 M diethanolamine, 1 mM MgCl_2_, 1 mg ml^−1^ BSA, 24 mM *p*-nitrophenylphoshate, pH 9.8). After mixing and incubation at 37°C for 10 min, the reaction was stopped by addition of 240 µl 0.5 N NaOH. The reaction was done in duplicate, and the absorbance was read at 405 nm.

### 4.9. Biotinylation of cell surface proteins and Notch detection

Pofut1^+/+^ and Pofut1^cax/cax^ SCDMs were plated in 100 mm tissue culture dishes, collected 2 days later with a cell scraper and washed three times with PBS (pH 8). For each of the cell lines, 7 × 10^6^ cells were incubated in 600 µl PBS (as a control) or PBS containing 1.66 mM EZ-link sulfo-*N*-hydroxysuccinimide-biotin (sulfo-NHS-biotin; Pierce Chemical Company, Rockford, IL) at 4°C for 45 min. The biotinylation reagent was then removed by centrifugation, and cells were washed three times with 1 ml of 100 mM glycine-PBS (pH 8) for 5 min to quench the biotinylation reaction. After cell lysis for 45 min at 4°C in 50 µl RIPA buffer, supernatants were recovered by centrifugation (12 000*g* for 10 min) at 4°C, and 400 µg soluble total proteins (input) were incubated overnight at 4°C with 50 µl avidin agarose (Pierce Chemical Company) previously equilibrated with RIPA buffer. Unretained proteins were recovered by centrifugation at 200*g* for 5 min, and avidin agarose was washed three times with diluted RIPA buffer for 20 min at 4°C. Retained biotinylated proteins were eluted from the Pierce avidin agarose by boiling for 5 min in the presence of 40 µl SDS–PAGE sample Laemmli buffer under reducing conditions. Amounts of loaded proteins were 25 µg for samples corresponding to input and unretained proteins. For eluted proteins, 15 µl supernatant from boiled avidin agarose was loaded. All samples were subjected to SDS–PAGE using 6% or 12% running gels and then transferred to Immun-Blot^®^ PVDF membranes (Bio-Rad, CA). After membrane blocking as previously described, a 1 : 500 dilution of an anti-NOTCH1 antibody (H-131) raised against amino acids 20–150 within the extracellular domain of human NOTCH1 (Santa Cruz Biotechnology, Santa Cruz, CA) was used to detect NOTCH1 at the SCDM cell surface. A 1 : 500 dilution of anti-pan-cadherin (Sigma-Aldrich Corp.) antibody was used to control the correct separation of membrane proteins. Anti-GAPDH (1 : 2000) was used to control that cytosolic proteins such as GAPDH were not biotinylated unlike membrane proteins at the cell surface.

### 4.10. Binding assays

Binding assays were performed either with adherent cells seeded at confluence in 12-well plates or with suspension cells placed in microcentrifuge tubes, as previously described [66]. About 500 000 adherent cells per well from Pofut1^+/+^ and Pofut1^cax/cax^ SCDMs were washed once with 2.5 ml HBAH and incubated at room temperature for 90 min with 500 µl crude supernatants from transfected COS-7 cells containing either Dll1-AP, Jag1-AP or Ctrl-AP at the same concentrations. The quantities of Dll1-AP, Jag1-AP and Ctrl-AP were normalized based on their AP activity. After gentle shaking every 30 min, cells were washed for 5 min six times with 2.5 ml HBAH. Cells were then lysed in two steps with a total volume of 200 µl TT buffer (10 mM Tris–HCl, 1% Triton X-100, pH 8), and lysates were centrifuged at 16 000*g* for 5 min. After heat inactivation of cell lysates in a 65°C water bath for 10 min and then incubation on ice for 15 min, we performed AP assay as described above to evaluate specific binding of the fusion proteins, considering Ctrl-AP as a negative control.

Owing to low amount of protein for Jag1-AP in medium from transfected COS7 cells compared with that for Dll1-AP and undetectable binding of Jag1-AP to adherent cells with crude supernatants, we performed binding assays with concentrated proteins from supernatants. Suspension cells were thus used instead of adherent cells to avoid important cell detachment that we had observed when using concentrated supernatants. The same protocol as above was performed with 200 000 suspension cells, previously washed with HBAH and incubated in the presence of 100 µl of concentrated supernatants for Ctrl-AP, Dll1-AP and Jag1-AP assayed at the same concentrations (40 < A^405 nm^ < 160), based on the AP assay. Washes were performed six times for 5 min with 500 µl HBAH at a low speed spin for 1 min at 2000*g* in a microcentrifuge. Cells were then lysed in 75 µl TT buffer and centrifuged at 16 000*g* for 5 min prior to heat inactivation and AP assay.

### 4.11. Immunofluorescent staining of tissue sections and cells

Dissected skeletal muscles (*Quadriceps*, *Gastrocnemius*, *Tibialis* and *Soleus*) from six Pofut1^+/+^ and Pofut1^cax/cax^ mice at three different ages (5, 12 and 24 weeks) were frozen in liquid nitrogen-cooled isopentane and stored at −80°C before being sectioned. Cryosections (10 µm) were thawed at room temperature and air-dried. Cryosections and SCDMs in primary cultures from Pofut1^+/+^ and Pofut1^cax/cax^ mice were fixed for at least 15 min with 4% paraformaldehyde (PFA) in PBS and washed three times in PBS. Only cells were permeabilized for 30 min at 4°C with a buffer pH 7.4 containing 20 mM HEPES, 300 mM sucrose, 50 mM NaCl, 3 mM MgC1_2_ and 0.5% Triton X-100. Both cryosections and cells were blocked for 1 h at room temperature in two blocking buffers (BBs): BB1 (10% goat serum, 1% BSA and 0.1% Triton X-100 in PBS) and BB2 (5% fetal calf serum, 2% BSA and 0.2% Triton X-100 in PBS). Then, they were washed with 0.2% BSA in PBS and incubated with primary antibodies diluted in PBS with 1% BSA for 1 h at 37°C. After three washes with PBS, 0.2% BSA, 0.1% Tween-20, slides were incubated for 15 min at 37°C with fluorescent conjugated secondary antibodies diluted 1 : 1000 in PBS 1% BSA as follows: Alexa Fluor^®^ 488 goat anti-mouse IgG (H+L) (Invitrogen, Thermo Fisher Scientific) for anti-MyHC (1 : 2000; Sigma-Aldrich Corp.) and anti-PAX7 (1 : 500; Developmental Studies Hybridoma Bank, University of Iowa, IA), Alexa Fluor^®^ 488 rabbit anti-goat IgG (H+L) (Invitrogen, Thermo Fisher Scientific) for anti-DESMIN (1 : 100; Santa Cruz Biotechnology) or Alexa Fluor^®^ 546 F(ab')2 fragment of goat anti-rabbit IgG (H+L) (Invitrogen, Thermo Fisher Scientific) for anti-LAMININ (1 : 500; Sigma-Aldrich Corp.) and anti-MyoD (1 : 1000; Santa Cruz Biotechnology). Staining was completed with three PBS washes and incubation for 5 min at room temperature in 1 µg ml^−1^ DAPI solution to label cell nuclei. Cells and cryosections were rinsed five times with PBS, mounted with Mowiol 4–88 mounting medium and sealed with glass coverslips.

For cryosections, data were analysed from 12 randomly chosen fields in each of 12 cryosections per muscle for each animal (six mice per genotype and per age) by using an epifluorescence microscope (Leica DMI4000B MM AF Imaging System) powered by MetaMorph (Universal Imaging Corp., Downingtown, PA) equipped with a 40× objective. Triple immunostaining (LAMININ/DAPI/PAX7) revealed the myofibre surface area (LAMININ^+^), all myonuclei (DAPI^+^) and specifically satellite cells (PAX7^+^) located peripherally. Mean fluorescence intensity of captured images was analysed by ImageJ. We converted each image into a binary one, then collected the pixel values (one pixel on ImageJ = 0.47 µm^2^ on Metamorph) and calculated the total area of each field in µm^2^. By manual counting, we determined the total number of myofibres, myonuclei and satellite cells on cross-sectional field. The mean myofibre area was obtained by dividing the total area of the field (µm^2^) by total number of myofibres. Then, the number of nuclei per myofibre was calculated as well as the number of Pax7+ satellite cells per myofibre, as recently published cryosection analyses [67].

For fusion indexes of SCDMs, data were analysed from 12 randomly chosen fields per well in duplicate for three independent experiments (*n* = 3). Images for fusion indexes were obtained by the same method as described above. Fusion index was calculated by dividing the number of myonuclei (DAPI+) contained in MyHC-expressing myotubes by the total number of myonuclei. To count the different cell populations expressing PAX7 and/or MYOD in Pofut1^+/+^ and Pofut1^cax/cax^ SCDMs, data were analysed from 12 randomly chosen fields per well in triplicate (*n* = 3).

### 4.12. Statistical analysis

All experiments were performed in biological triplicates and results are reported as the means ± s.e.m. Statistical comparisons were performed using two-tailed *t-*tests implemented in Prism, v. 5.03 (GraphPad Software, Inc., San Diego, CA). A *p-*value of 0.05 or less was considered statistically significant.

## Supplementary Material

Characteristics of skeletal muscles from 5, 12 and 24 week old Pofut1+/+ and Pofut1cax/cax mice.
