# Supplementary material for: Reduced Notch signalling leads to postnatal skeletal muscle hypertrophy in Pofut1cax/cax mice
Source: Open Biol. 2016 Sep 14;6(9):160211. doi: 10.1098/rsob.160211 (PMC5043585; doi:10.1098/rsob.160211)
Supplement: Characteristics of skeletal muscles from 5, 12 and 24 week old Pofut1+/+ and Pofut1cax/cax mice. [file rsob160211supp1.pdf]

SUPPLEMENTARY DATA

		Quadriceps	*Gastrocnemius*	*Tibialis*	*Soleus*	
**Muscle weight/Body weight ratios (%)**	**+/+**	1.11 ± 0.06	0.79 ± 0.03	0.33 ± 0.02	0.14 ± 0.01	**5 weeks**
	**cax/cax**	1.35 ± 0.03**	1.08 ± 0.06**	0.53 ± 0.04*	0.30 ± 0.02*	
	**+/+**	1.06 ± 0.04	1.10 ± 0.04	0.29 ± 0.01	0.15 ± 0.01	**12 weeks**
	**cax/cax**	1.31 ± 0.06 **	1.48 ± 0.04 **	0.43 ± 0.03 *	0.29 ± 0.01 *	
	**+/+**	0.98 ± 0.07	0.75 ± 0.03	0.26 ± 0.01	0.15 ± 0.00	**24 weeks**
	**cax/cax**	1.30 ± 0.03 **	1.01 ± 0.03 **	0.43 ± 0.03 *	0.27 ± 0.01 *	
**Myofibre mean area $(x10^3 \mu m^2)$**	**+/+**	2.06 ± 0.12	1.61 ± 0.11	1.88 ± 0.13	1.48 ± 0.11	**5 weeks**
	**cax/cax**	2.69 ± 0.14**	2.09 ± 0.04**	2.72 ± 0.10 ***	1.98 ± 0.08**	
	**+/+**	2.56 ± 0.08	2.57 ± 0.09	2.03 ± 0.08	1.77 ± 0.03	**12 weeks**
	**cax/cax**	3.34 ± 0.09 **	3.19 ± 0.04 **	2.83 ± 0.02 ***	2.95 ± 0.06 ***	
	**+/+**	2.86 ± 0.08	2.48 ± 0.06	2.01 ± 0.06	1.81 ± 0.11	**24 weeks**
	**cax/cax**	3.80 ± 0.15 **	3.11 ± 0.10 **	2.84 ± 0.17 *	2.55 ± 0.11 **	
**Nuclei/Myofibre**	**+/+**	3.03 ± 0.19	2.03 ± 0.43	2.79 ± 0.18	2.73 ± 0.25	**5 weeks**
	**cax/cax**	4.10 ± 0.22**	3.20 ± 0.20**	4.32 ± 0.21**	3.91 ± 0.14**	
	**+/+**	2.72 ± 0.06	2.98 ± 0.10	3.51 ± 0.18	2.69 ± 0.09	**12 weeks**
	**cax/cax**	3.90 ± 0.25 **	4.55 ± 0.04 **	4.88 ± 0.12 **	5.26 ± 0.26 ***	
	**+/+**	2.52 ± 0.06	1.08 ± 0.15	2.30 ± 0.06	3.04 ± 0.20	**24 weeks**
	**cax/cax**	3.67 ± 0.10 **	1.96 ± 0.16 **	3.41 ± 0.06 **	4.34 ± 0.11 **	
**Myofibres/Field**	**+/+**	234.14 ± 10.97	301.50 ± 11.25	296.56 ± 11.64	384.55 ± 10.16	**5 weeks**
	**cax/cax**	231.48 ± 15.27	293.83 ± 8.86	281.25 ± 11.59	356.05 ± 13.48	
	**+/+**	174.46 ± 7.39	191.86 ± 7.57	205.63 ± 17.45	233.07 ± 24.29	**12 weeks**
	**cax/cax**	177.06 ± 7.24	206.89 ± 20.22	212.37 ± 4.90	243.49 ± 21.93	
	**+/+**	167.64 ± 10.86	211.93 ± 8.46	195.03 ± 2.55	229.75 ± 14.46	**24 weeks**
	**cax/cax**	158.23 ± 6.70	218.06 ± 20.71	209.21 ± 9.46	223.29 ± 09.21	
**Satellite Cells/Myofibre**	**+/+**	0.068 ± 0.004	0.042 ± 0.004	0.033± 0.002	0.029 ± 0.002	**5 weeks**
	**cax/cax**	0.031 ± 0.002***	0.025 ± 0.003**	0.020 ± 0.001**	0.019 ± 0.002 **	
	**+/+**	0.054 ± 0.008	0.068 ± 0.002	0.046 ± 0.002	0.021 ± 0.002	**12 weeks**
	**cax/cax**	0.016 ± 0.002 ***	0.009 ± 0.001 ***	0.009 ± 0.005 **	0.005 ± 0.001 **	
	**+/+**	0.063 ± 0.02	0.034 ± 0.01	0.052 ± 0.002	0.031 ± 0.01	**24 weeks**
	**cax/cax**	0.015 ± 0.004 *	0.023 ± 0.009	0.023 ± 0.001 ***	0.004 ± 0.002 *	

Table S1. Characteristics of skeletal muscles from 5, 12 and 24 week old Pofut1$^{+/+}$ and Pofut1$^{cax/cax}$ mice. In addition to muscle weight/body weight ratios, myofibre mean area, number of nuclei per myofibre, number of myofibres per field and number of satellite cells per myofibre were determined for skeletal muscles with fast-twitch (*Tibialis*), slow-twitch (*Soleus*) or mixed (*Quadriceps*, *Gastrocnemius*) myofibres. Mean ± SEM are shown (t-test two-tailed, with a significance level of *: p<0.05, **: p<0.01, ***: p<0.001).

Al Jaam B, Heu K, Pennarubia F, Segelle A, Magnol L, Germot A, Legardinier S, Blanquet V, Maftah A. 2016. Reduced Notch signalling leads to postnatal skeletal muscle hypertrophy in Pofut1$^{cax/cax}$ mice. Open Biol. 6: 160211. http://dx.doi.org/10.1098/rsob.160211
